# Decellularized fennel and dill leaves as possible 3D channel network in GelMA for the development of an *in vitro* adipose tissue model

**DOI:** 10.3389/fbioe.2022.984805

**Published:** 2022-10-31

**Authors:** Francesca Grilli, Matteo Pitton, Lina Altomare, Silvia Farè

**Affiliations:** ^1^ Before Lab, Department of Chemistry, Materials and Chemical Engineering “G. Natta”, Politecnico di Milano, Milan, Italy; ^2^ INSTM, National Consortium of Materials Science and Technology, Local Unit Politecnico di Milano, Milan, Italy

**Keywords:** gelatin, vascularization, *in vitro* model, plant derivatives, decellularization, adipose tissue

## Abstract

The development of 3D scaffold-based models would represent a great step forward in cancer research, offering the possibility of predicting the potential *in vivo* response to targeted anticancer or anti-angiogenic therapies. As regards, 3D *in vitro* models require proper materials, which faithfully recapitulated extracellular matrix (ECM) properties, adequate cell lines, and an efficient vascular network. The aim of this work is to investigate the possible realization of an *in vitro* 3D scaffold-based model of adipose tissue, by incorporating decellularized 3D plant structures within the scaffold. In particular, in order to obtain an adipose matrix capable of mimicking the composition of the adipose tissue, methacrylated gelatin (GelMA), UV photo-crosslinkable, was selected. Decellularized fennel, wild fennel and, dill leaves have been incorporated into the GelMA hydrogel before crosslinking, to mimic a 3D channel network. All leaves showed a loss of pigmentation after the decellularization with channel dimensions ranging from 100 to 500 µm up to 3 μm, comparable with those of human microcirculation (5–10 µm). The photo-crosslinking process was not affected by the embedded plant structures in GelMA hydrogels. In fact, the weight variation test, performed on hydrogels with or without decellularized leaves showed a weight loss in the first 96 h, followed by a stability plateau up to 5 weeks. No cytotoxic effects were detected comparing the three prepared GelMA/D-leaf structures; moreover, the ability of the samples to stimulate differentiation of 3T3-L1 preadipocytes in mature adipocytes was investigated, and cells were able to grow and proliferate in the structure, colonizing the entire microenvironment and starting to differentiate. The developed GelMA hydrogels mimicked adipose tissue together with the incorporated plant structures seem to be an adequate solution to ensure an efficient vascular system for a 3D *in vitro* model. The obtained results showed the potentiality of the innovative proposed approach to mimic the tumoral microenvironment in 3D scaffold-based models.

## 1 Introduction

Breast cancer is one of the main types of tumors affecting women worldwide, diagnosed in one out of eight patients, and one of the principal causes of death (Mahmoudzadeh and Mohammadpour, 2016). Breast tumors develop in a tissue microenvironment containing extracellular matrix (ECM), adipocytes, stromal cells, and blood vessels. The realization of a suitable model, to be used for the *in vitro* cancer study is highly desirable. For the realization of this model, the environment-mimicking three-dimensional (3D) cultures represent an interesting alternative to the complex host environments of *in vivo* models or to the simple, but limited, two-dimensional (2D) *in vitro* models. In fact, 3D models can recapitulate *in vitro* the adipose tissue feature with the ultimate goal to reduce, refine, and replace (i.e., 3R’s principle) animal models for the investigation of physio-pathological dynamic in human diseases, allowing for a scalable and reproducible platform.

Among the 3D *in vitro* models, spheroids are extensively used to mimic acinar breast structures and allow the formation of epithelial cancer in a scaffold-free environment (Ryu et al., 2019). They allow the study of morphogenetic processes, interactions of between glandular epithelial cells and myoepithelial cells, and specific spatial signaling, that cannot be evaluated in traditional 2D cultures (Rijal and Li, 2016). However, spheroids fail in recapitulating the complete characteristics of the tumor microenvironment (Zhang et al., 2016). Another possible approach is the use of a fat-on-a-chip, that allows for the realization of a microfluidic platform where an environment with multiple compartments can simultaneously investigate the synergies when complex processes occur (McCarthy et al., 2020). Anyway, only few papers can be found in the literature, in which a substrate mimicking the adipose tissue is used for cell culture with the aim of mimicking the breast cancer microenvironment (Zhang et al., 2015; Bender et al., 2020; Fitzgerald et al., 2020; Yang et al., 2021).

As regards 3D scaffold-based models, they are under investigation for the development of systems able to mimic cancer tissue and study effective therapeutic approaches against cancer (Lugert et al., 2018). To successfully study the pathophysiology of human breast cancer in 3D systems three basic requirements can be highlighted (Kim et al., 2004): 1) co-culture of different cell types allowing the exchange of growth factors or other biological biomolecules and specific cell-cell interactions (e.g., cancer cells versus stromal cells, endothelial cells, fibroblasts and, immunocompetent cells); 2) design and realization of a synthetic ECM, to provide mechanical stability and to closely mimic the 3D architecture of the *in vivo* conditions; 3) nutrients and biological biomolecules, to promote cell differentiation and proliferation. Many synthetic and natural polymeric materials can be used for the realization of 3D scaffold-based *in vitro* model (Gibler et al., 2021). Among them, some examples are represented by collagen (Sims-Mourtada et al., 2014), chitosan (Anil-Inevi et al., 2020), alginate (Grolman et al., 2015), (Vinson et al., 2017), fibrin (Yang et al., 2021), (Böttcher-Haberzeth and Biedermann, 2019), (Paek et al., 2019), decellularized adipose tissue (Cheung et al., 2014), (Mohiuddin et al., 2019), polyethylene glycol/chitosan blend (Tsao et al., 2014), and collagen/chitosan blend (Mahmoudzadeh and Mohammadpour, 2016). In the last decade, large attention has been focused on crosslinked gelatin (Colle et al., 2020), (Contessi Negrini et al., 2019), in particular on methacrylated gelatin (GelMA) hydrogels (Wang et al., 2015), (Campiglio et al., 2019). In fact, gelatin-based hydrogels show excellent thermostability and are able to support cell activities since they naturally contain RGD peptides.

The development of 3D scaffold-based vascularized tumor models is a highly desired goal in regenerative medicine. In fact, compared to 2D models, they could exhibit, better biomimetic properties and functionality, providing the ability to accurately mimic tissue composition, architecture, and vascularization, even in the case of a tumor microenvironment. Actually, in a 3D environment, without the proximity to perfused micro-vessels (i.e., 100–200 μm), which provide essential nutrients and growth factors and ensure oxygen and waste transportation, the cells would encounter death (Song et al., 2014). However, most of the 3D scaffold-based models for tumor adipose tissue reported in the scientific literature are still simplistic in terms of the vascular structure of the cancer tissue. The design of a 3D adipose tissue cancer model should include a greater stiffness and a more irregular capillary network, compared to the healthy one, in order to characterize the carcinogenic mass tissue. However, modeling this aberrant vascularization is the main obstacle to the 3D model realization because reproducing the structure and dimensions (5–10 μm) of the capillary network is not easy. Therefore, the appropriate design of the 3D scaffold-based vascularized model, in terms of distribution and size of the channel network, still remains an open question. Different strategies are proposed in the literature to promote the realization of a channel network in 3D scaffolds, that can mimic the *in vivo* tissue vascularization, regardless of the final application (Lovett et al., 2009; Datta et al., 2017; Tomasina et al., 2019; Lopes et al., 2021). Among them, a classical approach for the vascularization in the 3D scaffolds considers the stimulation of the vasculature development within the construct by using growth factor delivery systems or a co-culture with endothelial cells. In fact, by combining different growth factors (e.g., VEGF, BFGF), a cross-talking among cells is promoted, enabling the sprouting of new vessels (Lopes et al., 2021), (Tomasina et al., 2019). In this approach, the scaffold features, e.g., stiffness, density, viscoelasticity, and crosslinking degree are important to enable the formation of vascular networks, since cells can exhibit different behavior depending on the stiffness and chemical stimuli in the micro-environment. In addition, this approach does not allow the generation of a reproducible vascular network. On the other hand, bioprinting is a fast method to deposit cells and materials in a precise and reproducible manner. This is a promising approach for the vascularization of the scaffolds, but it represents an emerging technology that requires optimization depending on the specific materials, cells, and requirements for the tissue to be regenerated or modelized (Kolesky et al., 2016; Laschke and Menger, 2016; Mandrycky et al., 2016; Saberianpour et al., 2018). The printing of a fugitive ink in a 3D shape resembling the tumoral vascular network represents an alternative use of the printing technique. In this approach, the channel network can be printed with a fugitive ink and embedded in a biocompatible cell laden hydrogel to build perfusable regular channel networks, once the fugitive ink is washed away (Contessi Negrini et al., 2019), (Miller et al., 2012). However, these strategies do not demonstrate the complete mimicking of native 3D vasculature, also because microvasculature (i.e., diameter lower than 10 μm) cannot be obtained by the 3D printing technique (Gershlak et al., 2017).

In the last years, great attention has been paid to bio-inspired approaches, in particular, taking advantage of the decellularization techniques for tissues and organs (Ott et al., 2008). To overcome problems arising in the use of decellularized human tissue (Sullivan et al., 2014), (Gilbert et al., 2006), a possible approach considers cross-kingdom. In fact, even if plants require different mechanisms for the transportation of fluids in their body, natural similarities are detectable in the vascular structure of plant and mammalian tissues, which can be exploited for the fabrication of a microvascular network with dimensions comparable with those of human capillaries (5–10 μm) (Ott et al., 2008). Several examples of decellularized plants and fruits are reported in the literature (Harris et al., 2021), such as spinach and parsley leaves to obtain perfusable structures (Ott et al., 2008), leek for the fabrication of potential biomaterials (Toker et al., 2020), spinach and chive as possible structures for kidney tubule (Jansen et al., 2021), apple, carrots, and celery to support adhesion and proliferation of preadipocytes, osteoblasts, muscle cells, respectively (Contessi Negrini et al., 2020a). However, to our knowledge, no investigation of the possible use of decellularized plants or fruits for mimicking a 3D vasculature in a 3D *in vitro* model has been reported in the literature.

The aim of this work is the investigation of the possible realization of an *in vitro* 3D scaffold-based model of adipose tissue, by incorporating decellularized 3D plant structures within the scaffold. In particular, in order to create an adipose matrix capable of mimicking the composition of the adipose tissue, methacrylated gelatin (GelMA), UV photo-crosslinked, was selected. Decellularized fennel, wild fennel and, dill leaves have been incorporated into the hydrogel to mimic a 3D channel network. The obtained 3D scaffolds were characterized by a physical, mechanical, and *in vitro* biological point of view, to investigate the suitability of the structures as a 3D model of pathological and vascularized, adipose tissue.

## 2 Materials and methods

### 2.1 Materials

All the materials were purchased from Sigma Aldrich unless differently specified. Fresh fennel, wild fennel and, dill leaves were acquired from the same chain store and stored at 4°C for maximum 5 days prior to decellularization.

### 2.2 Adipose matrix

#### 2.2.1 Synthesis of methacrylated gelatin

GelMA was used to mimic the adipose matrix in the 3D model, and was prepared as reported in the literature (Nichol et al., 2010), (Lim et al., 2016), and previous studies (data not shown). Briefly, type A gelatin from porcine skin (10% w/v) was dissolved in Dulbecco’s Phosphate Buffered Saline (DPBS) under stirring at 50°C. Methacrylic anhydride (MA, 8% v/v), was dropped in the gelatin solution at 50°C and stirred for 3 h. Then, the solution was diluted 5 times with DPBS, dialyzed in cellulose tubes (MW cut-off: 12–14 kDa) in distilled water for 1 week, and freeze-dried (LIO5P 129, 5 Pa) for 72 h.

#### 2.2.2 Methacrylated maiuscolo gelatin crosslinking

For the crosslinking, GelMA solution in DPBS (10% w/v) and the photoinitiator Irgacure 2,959 (2-hydroxy-4′-2-hydroxyethoxy)-2-methylpropiophenone) dissolved in ethanol (10% w/v) were separately prepared. Irgacure was then added to GelMA solution (0.05% v/v). Then, the solution was poured into PDMS molds (diameter = 10 mm, h = 3 mm), and placed under UV light (λ = 365 nm), at a distance of 3 cm from the light source for 150 s.

### 2.3 Channel network

#### 2.3.1 Leaf decellularization

**FIGURE 1:**
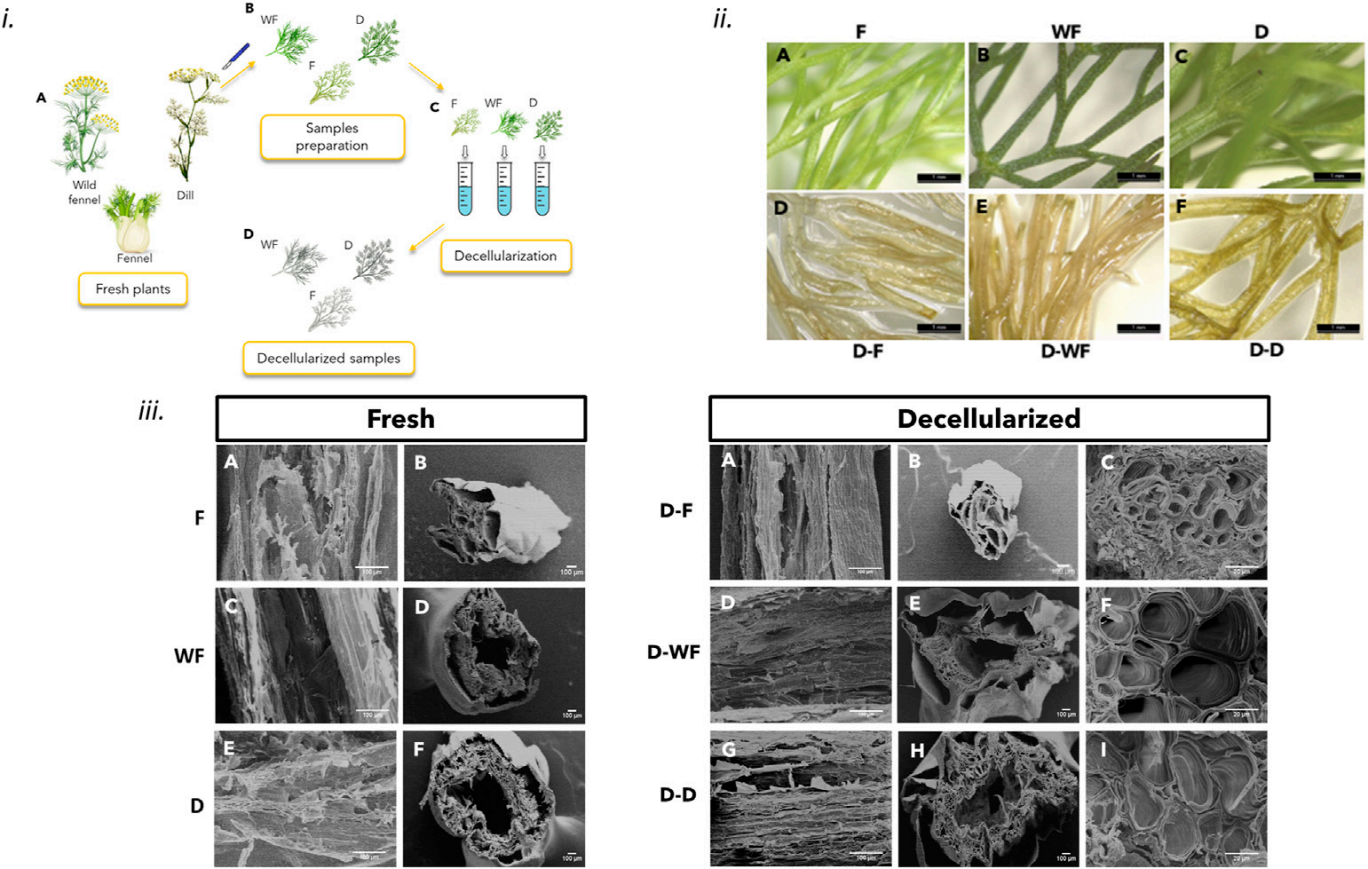
**i)** Schematic procedure for obtaining decellularized leaves from fresh plants: A) fresh plants; B) samples cut to obtain the leaf samples; C) decellularization process; D) decellularized leaf samples. **ii)** Stereomicroscope images: before (A,B,and C) and after (D,E,and F) the decellularization treatment for samples of (A,D) fennel (F), (B,E) wild fennel (WF) and (C,F) dill (D); scale bar: 1 mm **iii)** SEM images: before (Fresh) and after (Decellularized) of F, WF, and D: axial cross-section of the stem (Fresh: A,C,and E; Decellularized: A,D,and G); transversal cross-section of the stem (Fresh: B,D,F; Decellularized: B,E,H) high magnification of the transversal cross-section of the stem’s walls (Decellularized: C,F,I). Scale bar: 100 μm and 20 μm (C,F,I).

For mimicking the vascular network, three types of plants were selected (Figure 1-iA): cultivated fennel (F) and wild fennel (WF), *Foeniculum vulgare*, and dill (D), *Anethum graveolens*. Samples were obtained by cutting the ends of the leaves, using a scalpel, in order to obtain branched structures of about 2–3 cm in length (Figure 1-iB). The edge of the leaves has been cut to obtain open segments, promoting the infiltration of the decellularization solution inside the branches of the plant leaves. Decellularization (Figure 1-iC) was performed as reported in a previously optimized protocol (Contessi Negrini et al., 2020a), (Hickey et al., 2018). Samples were immersed in 0.1% w/v sodium dodecyl sulfate (SDS) for 48 h, under stirring at 180 rpm. After 24 h, sonication at 40°C for 5 min was performed, and the SDS step was repeated for a second time. Then, the samples were washed for 24 h in 100 mM CaCl_2_ solution for the SDS removal. Fresh samples (F, WF, D) and decellularized (D-F, D-WF, and D-D) samples were immersed in DPBS with 1% penicillin/streptomycin and 1% amphotericin B, under stirring at 140 rpm for 3 h. Finally, the decellularized samples (D-leaf) were immersed in 70% ethanol, stirred at 140 rpm for 1 h, frozen at −20°C, and freeze-dried for 48 h (Figure 1-iD).

#### 2.3.2 Morphological characterization

Morphological analyses were performed in order to provide a qualitative result of the decellularization protocol. In particular, the effect of decellularization on the samples’ macro and microstructure was assessed through the acquisition of stereomicroscope (Wild M8R) and Scanning Electron Microscope (SEM, Cambridge StereoScan 360R) images. Considering SEM images acquired for each decellularized leaf, 10 channels for each specimen were evaluated using Fiji ImageJ software in the area of the D-leaf reported in Supplementary Figure S2. In particular, larger channels (>100 nm) and smaller ones (<100 nm) were evaluated considering the SEM images acquired at ×100 and ×1000 magnification, respectively.

### 2.4 3D *in vitro* model

#### 2.4.1 Fabrication of the 3D model

**FIGURE 2:**
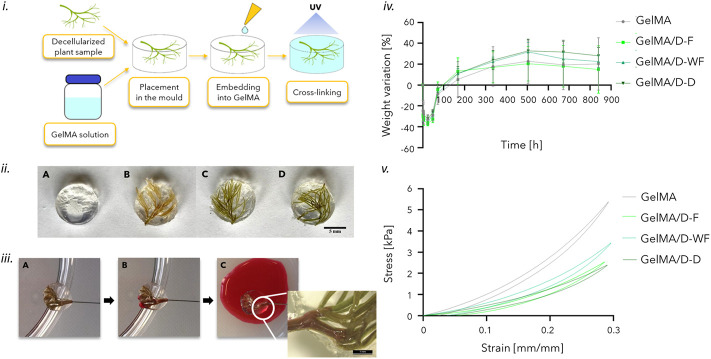
**i)** Scheme of the process to obtain the 3D scaffold-based model: insertion of the decellularized leaf (D-leaf) in the PDMS mold, dripping of the GelMA solution and UV light crosslinking. **ii)** Samples after the GelMA photocrosslinking: (A) GelMA, (B) GelMA/D-F, (C) GelMA/D-WF, and (D) GelMA/D-D; scale bar: 5 mm **iii)** Perfusion test on GelMA/D-F structures by injection of red dye: A) before the injection; B) after the complete filling of the hollow channel; a particular of the perfused structure: red dye is visible in the decellularized channels of the leaf branches. **iv)** Percentage weight variation curves of GelMA/D-leaf structures and GelMA samples. **v)** Representative stress-strain curves of GelMA/D-leaf structures and GelMA, obtained by mechanical compression tests.

For the fabrication of the 3D models, the decellularized and freeze-dried D-F, D-WF, and D-D leaf samples were inserted in PDMS molds (diameter = 10 mm, h = 3 mm). Then, 274 μl GelMA/photoinitiator solution (prepared as reported in Par. 2.2.2) were dropped onto the vegetal structure and crosslinked by UV light at a distance of 3 cm from the UV lamp for 150 s (Figure 2-i).

#### 2.4.2 Perfusion test

Perfusion tests were carried out to investigate the patency of the channels in the decellularized leaves, before and after incorporation into GelMA. A syringe pump (KD Scientific, KDS 100) was used with a Terumo 5 cc syringe connected to the decellularized structure sample (*n* = 3 for each type) *via* a 30 G needle, and inserted into the stem of the decellularized structure. The patency of the channels was then qualitatively assessed by injecting red food coloring at a flow rate of 1 and 2 ml/h.

#### 2.4.3 *In vitro* stability tests

The GelMA samples containing the decellularized leaves (*n* = 3 for each leaf) were subjected to a weight variation test; GelMA samples (*n* = 3) were used as a control to evaluate the influence of embedded plant structures on the crosslinking efficiency. Samples were incubated in an aqueous solution with 0.02% w/v NaN_3_, at 37°C and weighed at different time points (t_0_ = 0 h, t = 1, 2, 3, 4, 24, 48, and 72 h and every week up to 5 weeks). The percentage weight variation (*ΔW* %) was calculated using Equation (Mahmoudzadeh and Mohammadpour, 2016):

$$∆W\;\left[\%\right]=\frac{w_{t}-w_{0}}{w_{0}}\times 100$$ (1)

where: *w*_*0*_ is the weight at t_0_ and *w*_*t*_ is the weight at the considered time point.

#### 2.4.4 Mechanical characterization

Mechanical compression tests were performed using a Dynamic Mechanical Analyzer (DMA Q800, TA Instruments). Samples (*n* = 3, diameter = 10 mm, *h* = 3 mm) of GelMA/D-F, GelMA/D-WF, and GelMA/D-D structures, and GelMA used as control, were tested, after reaching the swelling plateau. The test was performed at 37°C by applying a hysteresis compression cycle. A preload force (F = 0.001 N) was applied, followed by a loading phase at a rate of 2.5%/min, down to −30% strain, and an unloading phase at a rate of 5%/min. From the stress/strain curves, elastic modulus, E (calculated as the slope in the 0%–5% strain range), stiffness, K (calculated as the slope in the 25%–30% strain range), maximum stress, σ_max_, residual deformation, ε_res_, and hysteresis area, H (calculated as area between the load and the unload curves) were obtained (Contessi Negrini et al., 2020a).

#### 2.4.5 *In vitro* biological test

Direct cytocompatibility tests were performed on GelMA/decellularized leaves (D-F, D-WF, D-D) samples and GelMA as control. 3T3-L1 preadipocyte murine cell line (ECACC No. 86052701) was selected to investigate the possible application of the structure as an *in vivo* 3D scaffold-based model. Cells (density = 1 × 10^6^ cells/ml) were embedded in GelMA/photoinitiator solution and 274 μl of GelMA/photoinitiator/cells were dripped in the PDMS molds, in which the decellularized leaves were previously positioned. GelMA samples with or without decellularized leaves were crosslinked by UV light. Cells seeded on TCPS were used as a control. Preadipocyte growth medium (Dulbecco’s Modified Eagle Medium, DMEM with 10% v/v fetal bovine serum, 1% penicillin/streptomycin, 1 mM sodium pyruvate, 10 mM HEPES, 4 mML-glutamine) was used, renewing every 2 days. AlamarBlue assay was performed at 1, 3, and 7 days after seeding, to quantitatively assess the metabolic activity of 3T3-L1 cells in the cell-laden samples (*n* = 3 for type). Briefly, at each timepoint, samples were incubated for 4 h in 1 ml AlamarBlue solution in culture medium. Then, 100 μl were transferred in triplicate in a 96-well plate and fluorescence was read by a spectrophotometer (GENios Plus Reader; λ_exc_ = 540 nm, λ_em_ = 595 nm). The metabolic activity of the cells encapsulated in the samples, compared to the cells on TCPS (control), was evaluated as Relative Fluorescence Unit (RFU), using Equation (Rijal and Li, 2016) and Equation (Zhang et al., 2016). Samples were rinsed twice with PBS and incubated with fresh culture medium until the next timepoint.

$${RFU}_{embedded\;cells}={RFU}_{sample\;with\;cells}-{RFU}_{sample\;without\;cells}$$ (2)

$${RFU}_{cells\;\left(control\right)}={RFU}_{cells\;on\;TCPS}-{RFU}_{AlamarBlue}$$ (3)

Live/Dead staining was performed 1, 3, and 7 days after seeding, on GelMA/decellularized leaf samples (*n* = 2 for type) to qualitatively investigate the distribution of viable and dead cells in the 3D environment. Samples were incubated in the staining solution (10 μM propidium iodide and 2 μM calcein-AM in DMEM) for 40 min, washed three times with PBS, and immersed in culture medium without FBS. Images (*n* = 3) were acquired by fluorescence microscope (Olympus BX51W1) and analyzed by ImageJ software (NIH, Unites States). The percentage of viable cells in each acquired image was quantitatively measured using Equation (McCarthy et al., 2020), and the cell viability was obtained averaging the values derived from the three images:

$$cell\;viability\;\left[\%\right]=\frac{N_{live\;cells}}{N_{live\;cells}+N_{dead\;cells}}\times 100$$ (4)

**FIGURE 3:**
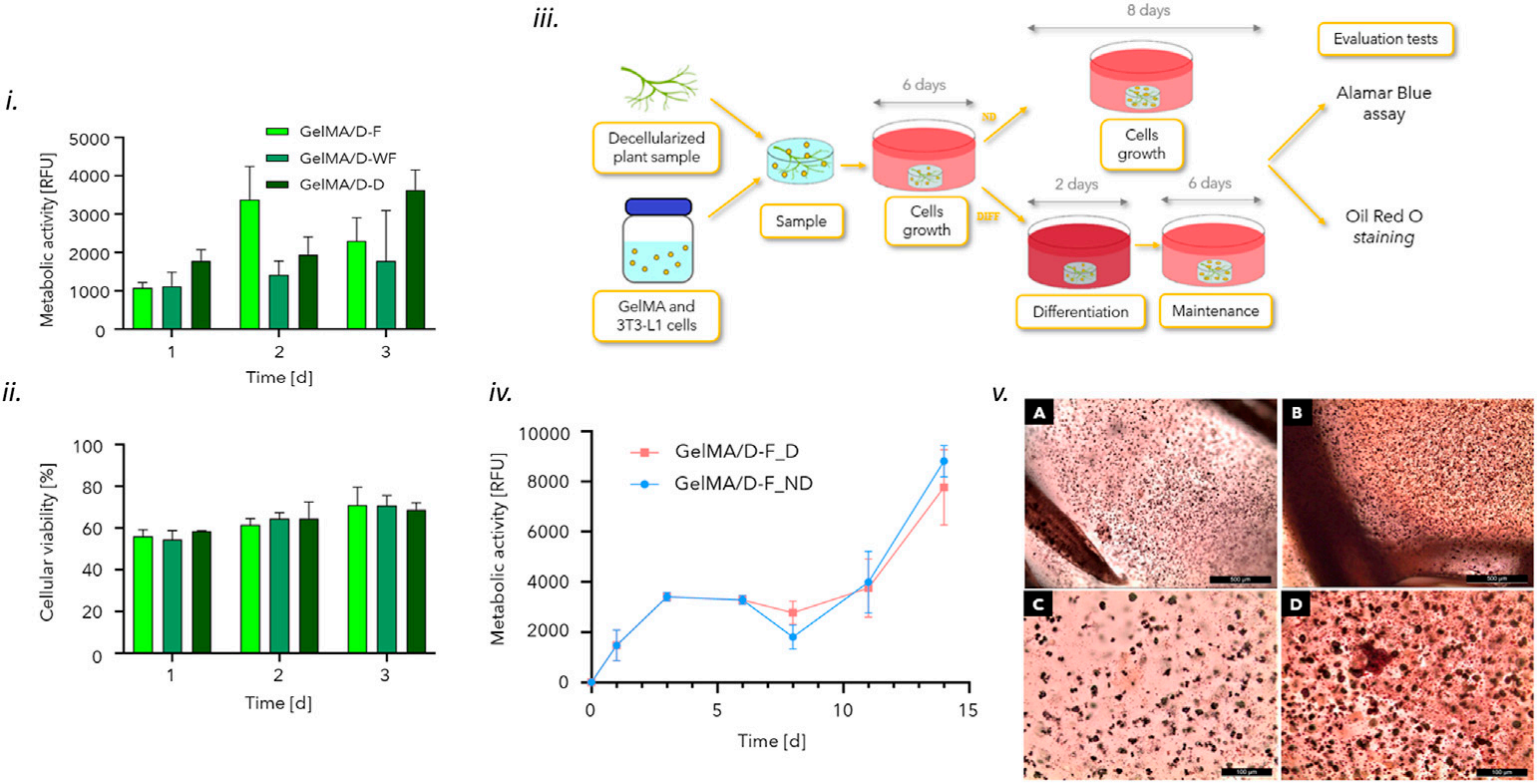
**i)** Metabolic activity (RFU) values at the considered timepoints for 3T3-L1 cells encapsulated in crosslinked GelMA/D-F, GelMA/D-WF, and GelMA/D-D. **ii)** Cell viability [%] obtained by Live/Dead staining up to 7 days of culture. **iii)** Scheme of 3T3-L1 cell seeding, growth and differentiation in the crosslinked GelMA/D-F structures. Cells were embedded in the hydrogel before crosslinking, and crosslinked samples were incubated in preadipocytes culture medium for 7 days; then, half of the samples were kept growing in preadipocytes growth medium, while half was induced to differentiation for 48 h in differentiation medium, replaced with a maintaining medium. **iv)** Metabolic activity (RFU) for 3T3-L1 cells embedded in crosslinked GelMA/D-F 1, 4, 6, 8, 11, and 14 days after seeding; after 6 days of culture, cells were kept in growth medium (GelMA/D-F_ND) or induced to differentiation by incubation for 48 h in differentiation medium and then in maintaining medium (GelMA/D-F_D). **v)** Oil Red O assay on GelMA/D-F structures induced to differentiation (A, C) and maintained in preadipocyte growth medium (B, D) after 14 days of culture. Scale bar: 500 μm (upper row) and 100 μm (bottom row).

Adipogenic differentiation tests (Figure 3-iii) were performed on GelMA/D-F samples, following the protocol described elsewhere (Contessi Negrini et al., 2020b). Cells (density = 1 × 10^6^ cells/ml) were embedded in GelMA/D-F and seeded on 24-well TCPS plates, as control, using preadipocyte growth medium, renewing every 2 days. After 6 days of culture, adipogenic differentiation was induced by culturing samples in differentiation culture medium for 48 h (DMEM with 10% v/v fetal bovine serum, 1% penicillin/streptomycin, 1 mM sodium pyruvate, 10 mM HEPES, 4 mML-glutamine, 1 μg ml^−1^ insulin, 0.5 mM 3-isobutyl-1-methylxanthine (IBMX), 1 μM dexamethasone (DEX), and 1 μM rosiglitazone). Then, differentiation-induced samples and cells seeded on the wells were kept in maintenance medium (composed by differentiation medium without IBMX, DEX, and rosiglitazone) up to 14 days of culture, renewed every 2 days up to 14 days. As a control, cell-laden samples and cells on the wells in which differentiation was not induced, were kept in growth culture medium for 14 days. The metabolic activity was evaluated by the AlamarBlue assay at 1, 3, 6, 8, 11, and 14 days after seeding, as previously described. After 14 days of culture, the adipogenic differentiation of 3T3-L1 cells embedded in the samples was investigated. Oil Red O was performed on GelMA/D-F structures, both differentiated and undifferentiated (*n* = 2) to evaluate the accumulation of lipid droplets after 14 days of culture. The staining solution was prepared by dissolving 300 mg of Oil Red O powder in 100 ml isopropanol overnight, then diluted 3:2 in distilled water (Contessi Negrini et al., 2020a). After 14 days of culture, samples were washed twice in DPBS, fixed by submersion in 4% w/v paraformaldehyde for 12 h, and washed twice with DPBS. Samples were incubated in 1 ml Oil Red O staining solution for 12 h and washed twice with DPBS prior to observation by optical microscopy (Leica DFC290).

### 2.5 Statistical analyses

Data are presented as mean and standard deviation. Statistical analysis was performed by *t*-test to compare two data sets or by one-way ANOVA test, with Tukey’s multiple comparison test, to compare more data sets. GraphPad Prism 8.0.27 software was used; the significance level was set at *p* < 0.05.

## 3 Results

### 3.1 Channel network

#### 3.1.1 Morphological characterization

The morphological analysis allowed to assess that the decellularization process performed did not damage the leaf structures for F, WF, and D. Stereomicroscope analysis (Figure 1-ii) showed the loss of pigmentation on the leaves following the decellularization treatment. In particular, during the treatment, all the samples initially assumed a browner color, then tended towards translucent white. This phenomenon is a typical result of the decellularization treatment, due to the removal of chlorophyll from the leaf tissue during the washing phases (Dikici et al., 2019) and it is more evident in the D-F samples than in D-WF and D-D samples. Moreover, the leaves of the three species have internal longitudinal marbling along their structure, which is preserved after decellularization.

SEM observation of the decellularized leaves showed the conservation of the structure compared to the fresh leaves (Figure 1-iii) and the presence of channels of various sizes (Figure 1-iii). A quantitative estimation of the channel diameter was performed starting from the high-magnification images (i.e., 1,000X) obtained on the cross-sections of the leaves (Figures 1-iiiC,F,I). The obtained values (Table 1, Supplementary Figure S1) showed that in the three types of leaves, the presence of channels (diameter: 100–500 μm) and microchannels (diameter: 3–35 μm) inside the walls of the branches is detectable. A different channel diameter distribution was observed for the D-leaves under evaluation; D-F showed smaller channel diameter (<5 μm), not found in the cross-section of D-WF and D-D that showed a higher quantity of channels in the diameter range 5–35 μm. The size of the microchannels (diameter = 3 μm) is comparable to those of human capillaries (5–10 μm), both in physiological and tumoral conditions (Gershlak et al., 2017), (Fung and Zweifach, 1971).

**TABLE 1:** Range of diameters of the channels to the three considered leaves (i.e., Fennel, Wild Fennel, Dill). The dimensions of the wider channels in the stems and those of the smaller channels in the outer branches have been reported.

Type of leaf	Larger channels [μm]	Smaller channels [μm]
Fennel	100–200	3–20
Wild Fennel	700–800	5–35
Dill	700–800	5–35

### 3.2 3D *in vitro* model

The protocols for the realization of the *in vitro* models made of GelMA and the embedded leaf of the different plant species are reported in Figure 2-i. Decellularized structures resulted in well-embedded and random organization in the GelMA hydrogel (Figure 2-ii).

#### 3.2.1 Perfusion tests

Through the perfusion tests, it was observed, at a qualitative level, the effective possibility of perfusing the 3D decellularized structures embedded in GelMA. The process of decellularization and incorporation of the leaves in the crosslinked GelMA have both contributed to and allowed the manufacture of a 3D network of functional channels, able to support the flow of fluid within the sample. From a qualitative point of view, the perfusion was consistent among the specimens tested for each type of plant leaves (i.e., D-D, D-F, and D-WF), and the perfusion appeared different among the three D-leaves. In particular, D-F leaves qualitatively were better perfused than the D-WF and D-D ones. Representative images of the performed perfusion tests are reported in Figure 2-iii. These images were acquired before and after perfusion, to investigate the patency of the leaf vascular system. The red dye, injected at a flow rate of 1 and 2 ml/h, filled the channels of the main stem, then distributed in some branches and exited the sample from the end of the branches (Supplementary Video S1).

#### 3.2.2 *In vitro* stability tests

The weight variation tests allowed for evaluating the swelling and possible degradation of the samples, immersed in an aqueous solution at 37°C, up to 35 days. The weight variation trends (Figure 2-iv) showed that the GelMA/D-leaf structures lost weight in the first 24 h, and reached the plateau at 168 h (i.e., *p* > 0.05 for each structure). The significant initial weight loss for all the samples between 0 and 72 h can be attributed to the leakage from the sample volume of the non-crosslinked gelatin macromolecules.

The weight variation curve of the GelMA samples, used as control, showed the same trend, demonstrating that the D-leaf structures embedded in GelMA before the crosslinking did not negatively affect the crosslinking efficiency. Hence, the presence of branched and 3D randomly organized decellularized leaves in the GelMA hydrogels did not significantly influence the UV-light crosslinking process. In fact, comparing the weight loss among the three GelMA/D-leaf and pristine GelMA no statistical difference can be highlighted at each time point. To confirm that, a gel fraction test was performed on GelMA and GelMA/D-F (SI-2). Comparing the gel fraction results at two different time points (i.e., 24 and 72 h) no statistical difference can be detected, evidencing a gel fraction of 82.29% ± 2.11% and 76.82% ± 1.21%, respectively for GelMA and GelMA/D-F after 72 h.

#### 3.2.3 Compression mechanical characterization

Compression tests were performed on crosslinked GelMA/D-leaf structures and pristine GelMA samples; the stress-strain curve for each analyzed sample was obtained (Figure 2-v) and mechanical parameters are reported in Table 2. A decrease in mechanical characteristics was found for all the samples containing plant structures compared to GelMA samples. In fact, compared to GelMA/D-leaf, pristine GelMA showed a higher slope which is correlated to a greater (*p* < 0.05) elastic modulus (Table 2, Supplementary Figure S3). However, the obtained elastic modulus values (3–9 kPa) for all the analyzed structures, are representative of adipose tissue, healthy (2–3 kPa), or at the first stages of tumor development (6–10 kPa) (Brandl et al., 2010). GelMA curve assumed a higher slope (*p* < 0.05) in the strain range considered for the stiffness, a higher value (*p* < 0.05) for σ_max_, representative of higher mechanical strength, and a higher (*p* < 0.05) hysteresis area value, related to the energy loss during the mechanical test, typical for viscoelastic materials. The lower mechanical behavior can be attributed to the fact that there is an irregular internal structure in the GelMA/D-leaf scaffold, including the presence of hollow channels inside the decellularized leaves (Ogushi et al., 2013).

**TABLE 2:** Values of the considered mechanical parameters obtained in the compression test performed on the four formulations considered. The average standard deviation of the parameter is shown in the table.

	GelMA	GelMA/D-F	GelMA/D-WF	GelMA/D-D
E [kPa]	9.32 ± 1.85^a,b,c^	4.27 ± 1.16^a^	5.68 ± 0.76^b^	3.55 ± 0.34^c^
K [kPa]	34.83 ± 4.42^a,b,c^	19.86 ± 3.06^a^	21.35 ± 0.97^b^	12.49 ± 1.67^c^
*σ*_max_ [kPa]	5.95 ± 0.90^a,b,c^	3.28 ± 0.62^a^	3.69 ± 0.19^b^	2.19 ± 0.18^c^
ε_res_ [%]	2.42 ± 0.28^c^	4.22 ± 0.87	3.43 ± 0.60	5.45 ± 0.81^c^
H [J/dm^3^]	0.65 ± 0.11^a,b,c^	0.34 ± 0.07^a^	0.39 ± 0.03^b^	0.23 ± 0.02^c^

Note: a, b, c, d, e, and f highlight the statistically significant differences (*p* < 0.05): a = GelMA vs. GelMA/D-F; b = GelMA vs. GelMA/D-WF; c = GelMA vs. GelMA/D-D; d = GelMA/D-F vs. GelMA/D-WF; e = GelMA/D-F vs. GelMA/D-D; f = GelMA/D-WF vs. GelMA/D-D.

### 3.3 *In vitro* biological test

#### 3.3.1 Cytocompatibility tests

*In vitro* biological tests were carried out on crosslinked GelMA/D-leaf specimens to verify the compatibility of vegetal structures once cells were embedded in the GelMA-based structure. In particular, direct cytocompatibility tests were carried out to verify that decellularized leaf structures did not release any potentially toxic substances resulting from the decellularization treatment, possibly affecting the behavior of encapsulated 3T3-L1 cells. AlamarBlue assay results showed comparable RFU values (*p* > 0.05) for the three GelMA/D-leaf structures at each timepoint (Figure 3-i). These results demonstrated that no difference between the cytocompatibility of the different leaves can be detected and that the involved decellularization reagents did not affect cell viability.

This trend was confirmed by the qualitative investigation of cell viability by the Live/Dead assay, that allowed verifying the adhesion and proliferation of 3T3-L1 cells on the outer surface of the D-leaves and in the whole bulk of the GelMA scaffold (Supplementary Figure S4). From the quantitative analysis of the results, no statistically significant differences were detected in cell viability comparing the structures with the different types of leaf (Figure 3-ii). Thus, the Live/Dead assay trend confirmed the results of the AlamarBlue test (Figure 3-i), demonstrating that the decellularization treatment and the D-leaves in GelMA are not cytotoxic and, moreover, that the process of crosslinking GelMA, in terms of photoinitiator, distance from the UV lamp, and time of exposure to UV light are not harmful to cell viability.

#### 3.3.2 Adipogenic differentiation tests

Since no cytotoxic effects were detected in the cytocompatibility test, comparing the three prepared GelMA/D-leaf structures, the ability of the samples to stimulate differentiation of 3T3-L1 preadipocytes in mature adipocytes was investigated. The GelMA/D-F structure was selected among the three 3D models, since no significant differences among the three types of plant were detected in all the tests previously carried out, at a physical, mechanical, and *in vitro* biological level.

AlamarBlue assay showed increasing RFU values during the 14 days of culture, comparable to the samples induced to adipogenic differentiation and those maintained in the preadipocyte culture medium (Figure 3-iv). In fact, an overlapping trend of the metabolic activity was detectable on cells embedded in GelMA/D-F differentiated and undifferentiated in adipocytes, during the 14 days of culture. Moreover, the metabolic activity of the cells encapsulated within the hydrogel showed a statistically significant increase (*p* < 0.05), for both types of culture (i.e., D vs. ND), between day 1 and 14, demonstrating the good viability of encapsulated cells and a trend to increase their metabolic activity. The images acquired after the Oil Red O staining showed a uniform and distributed cell organization in GelMA/D-F (Figure 3-v). In particular, GelMA/D-F_D seems to have a greater accumulation of lipid droplets, suggesting a better differentiation of the cells, by qualitative observation.

## 4 Discussion

For the realization of a 3D *in vitro* model of tumoral breast cancer adipose tissue, the components that have to be considered, are the adipose matrix, with its cellular and protein components, and the tortuous blood vessels. For the three types of leaves, 3D scaffolds embedding perfusable D-leaf were obtained and stability over time (about 5 weeks, i.e., test duration) was detected, with physical and mechanical characteristics comparable one each other. Furthermore, by comparing the behavior of such samples with those of control GelMA, the weight variation curves showed the same trend, demonstrating an equal crosslinking efficiency. Even though the mechanical characteristics of GelMA are greater, all the formulations nevertheless fall within the range of adipose tissue (Brandl et al., 2010). The *in vitro* biological tests, performed on the samples containing the three D-leaf, showed comparable results for all formulations, demonstrating the cytocompatibility of the D-leaf in the GelMA hydrogel as well as the decellularization treatment. In fact, an excellent ability of these samples to host the growth and proliferation of 3T3-L1 cells could be observed. On the other hand, as regards cell functionality, from the quantitative tests carried out after 14 days of culture (i.e., differentiation time = 8 days), no results were obtained in favor of adipogenic differentiation, found only at a qualitative level by Oil-red-O staining. The poor results achieved for all the analyzed formulations, with regard to cell differentiation, were related to a delay in the stimulation of the cells due to their encapsulation within the material (Ogushi et al., 2013). Future developments may focus on a deeper biological characterization to verify the expression of genes responsible (i.e., PPARγ) for the differentiation of preadipocytes encapsulated in GelMA/D-leaf.

In this work, particular attention has been paid to the vascular structures and, in particular, to the possibility of mimicking the structural and dimensional characteristics of cancer-associated blood vessels using an innovative approach. Decellularized plant structures were considered for this purpose due to the natural similarity of their microfluidic network to the human vascular one (i.e., structure and size). In fact, human vascular networks, both physiological and pathological, respond *in vivo* to angiogenic signals, which stimulate their growth to form capillary beds with complex branching, distributed over several planes and along random paths (Chan et al., 2012). These characteristics are difficult to replicate with modern 3D printing techniques, allowing for channel networks complex in terms of branching and interconnections, but generally very simple as geometries (e.g., regular tubes) and with high dimensions (e.g., diameter >100 μm). Instead, it is possible to notice a natural similarity between the plants and mammals vascularization, both able to support the flow of fluids and the transport of nutrients and other important biomolecules. Hence, the involvement of decellularized plant structures is a possible approach to the realization of 3D engineered constructs of vascularized tissue that are not replicable, instead, with most of the modern 3D printing techniques. In addition, cellulose, the main component of plant cell walls, is a polysaccharide widely studied as a biomaterial in regenerative medicine applications (Czaja et al., 2006; Müller et al., 2006; Zaborowska et al., 2010; Modulevsky et al., 2016). In this work, therefore, we have tried to investigate this possible new approach and to preliminary verify that decellularized leaves can actually serve as a structure to mimic a microchannels network in a 3D scaffold-based *in vitro* cancer model.

GelMA, obtained from gelatin type A, was chosen as the material for the realization of crosslinked 3D matrix mimicking the adipose tissue. This material is widely used in regenerative medicine for the regeneration and *in vitro* models of adipose tissue, because, once crosslinked, it is able to mimic its characteristics both in terms of protein composition and mechanical properties (Colle et al., 2020), (Ovsianikov et al., 2011; Huber et al., 2016; Kessler et al., 2017). For mimicking the vascularization network, cultivated and wild fennel, and dill, were selected based on their macroscopic structural similarity (e.g., branches), to human microcirculation. In fact, *in vivo*, larger vessels branch into smaller lateral channels, with diameters ranging from a few millimeters to about ten microns (Fung and Zweifach, 1971). The results obtained by SEM observation for the decellularized leaf cross-sections have shown that the microchannels present within these plant structures have diameters ranging from about 500 μm up to 3 μm, therefore in line with the size of blood vessels *in vivo* (Supplementary Table S1). The selected leaves were decellularized for the removal of cell material and DNA following a protocol well described in the literature and used for the decellularization of different types of plants (Ott et al., 2008), but never applied to fennel and dill. All the considered leaf tissues exhibited a loss of pigmentation after the decellularization treatment. A translucent appearance is typical of decellularized structures, both for the animal (Bühler et al., 2015) and plant tissues (Gershlak et al., 2017), (Fontana et al., 2017), (Adamski et al., 2018). Loss of pigmentation is due to the removal of chlorophyll from leaf tissue during washing steps (Dikici et al., 2019). In (Modulevsky et al., 2016), apple samples were observed, after treatment, by SEM, highlighting the architecture of the scaffolds in an acellular and highly porous 3D cellulose frame, and by hematoxylin-eosin staining, which allowed the qualitative assessment of the absence of native apple cells after decellularization. In (Adamski et al., 2018) spinach leaf samples were analyzed after decellularization to quantify DNA removal. The genomic DNA of the samples was isolated and quantified, allowing to verify a significant reduction in treated samples compared to no decellularized samples. In (Gershlak et al., 2017) and (Fontana et al., 2017) the colorimetric test performed to evidence the retention of genomic DNA and proteins in spinach leaf samples and parsley stems and roots showed that the decellularized structures had levels of remaining DNA that met an adequate level. These results reported in the literature well demonstrated the efficiency of using a protocol of decellularization based on the use of SDS for plant matrices. Hence, the selected decellularization protocol allowed for the preservation of the original structure both in the superficial and inner layers, maintaining the highly porous structure, with channels inside the wall that continue throughout the sample. For all these reasons, we decided to apply the protocol without further characterization.

It is important to highlight that, to our knowledge, no studies like the one here presented are reported in the literature. The novelty of the present work mainly concerns the use of decellularized plants in a scaffold. Even if different papers reported how the vascularization of leaves could mimic the human one, the decellularized structures are themselves used as a scaffold (Bilirgen et al., 2021). Apart from the use of different plants as a possible 3D scaffold, cardiac tissue engineering, osteogenesis, and bone tissue engineering, skeletal muscle, adipose tissue, and tendons, some researchers demonstrated the possible use of apple (Hickey et al., 2018), spinach leaves (Dikici et al., 2019), medicinal plant leaves (Thippan et al., 2019), *Camellia japonica* (Varhama et al., 2019), *Aptenia cordifolia* (Wang et al., 2020) to support blood vessel formation or vascularization. However, these structures have never been embedded in a 3D scaffold that could mimic the characteristic of the tissue to be regenerated or *in vitro* mimicked. The advantage of the here proposed approach allows for the three-dimensionality of the channel network that cannot simply be obtained by using 3D printing techniques. A new technique of free form reversible embedding of suspended hydrogels (FRESH) is also gaining attention in recent years, which would allow the printing not only in the x-y plane but also in the *z* axes (Holland et al., 2018). However, to date, only a few attempts have been made with it. Furthermore, even if FRESH allows the printing of complex and interconnected 3D structures (e.g., femur models and vessels with bifurcations) with high fidelity by embedding cells in the bioprinted hydrogel, no adipose cancer 3D scaffold-based models have been already proposed. Moreover, the possible limitation of the 3D printing technique in recapitulating the 3D vascular network concerns the difficulty in changing the diameter of the channel in the same network. In the past years, new techniques emerged to deposit simultaneously several materials that should be used as fugitive ink so to obtain a network with channels with different diameters or to directly bioprint structures embedding vascular cells. The multi-material 3D printing is possible as co-extrusion, multi-jet including side-by-side, co-axial bioprinting, core-shell bioprinting or gradient and consists in bioprinting more than one material in a single step. The possibility of reducing the diameter of the channel, like in the natural vascular network, can be obtained by using decellularized plants having a branched structure in a manner simpler than the use of fugitive inks.

## 5 Conclusion

Nowadays, the development of 3D scaffold-based *in vitro* models would represent a great step forward in cancer research, offering the possibility of predicting *in vitro* the potential *in vivo* response to targeted anticancer or anti-angiogenic therapies. In this work, we chose GelMA/D-leaf structures having a branched structure similar to the vascular network. Starting from these structures, we obtained 3D models showing adequate channel dimensions and mechanical properties adequate to mimic the adipose tissue microenvironment. A preliminary *in vitro* biological study showed that the encapsulated 3T3-L1 cells after an initial period of metabolic adaptation, were able to grow and proliferate in the structure, colonizing the entire microenvironment and starting to differentiate. The obtained results showed the potentiality of the innovative proposed approach, based on the use of natural plants for the vascularization network that well mimics the one of the tumoral microenvironment in 3D scaffold-based models.

## Data Availability

The raw data supporting the conclusion of this article will be made available by the authors, without undue reservation.

## References

[B1] Adamski M. Fontana G. Gershlak J. R. Gaudette G. R. Le H. D. Murphy W. L. (2018). Two methods for decellularization of plant tissues for tissue engineering applications. J. Vis. Exp. 135, 57586. 10.3791/57586 PMC6101437 29912197

[B2] Anil-Inevi M. Sağlam-Metiner P. Kabak E. C. Gulce-Iz S. (2020). Development and verification of a three-dimensional (3D) breast cancer tumor model composed of circulating tumor cell (CTC) subsets. Mol. Biol. Rep. 47 (1), 97–109. 10.1007/s11033-019-05111-z 31583566

[B3] Bender R. McCarthy M. Brown T. Bukowska J. Smith S. Abbott R. D. (2020). Human Adipose derived cells in two- and three-dimensional cultures: Functional validation of an *in vitro* fat construct. Stem Cells Int. 2020, 1–14. 10.1155/2020/4242130 PMC7303735 32587620

[B4] Bilirgen A. C. Toker M. Odabas S. Yetisen A. K. Garipcan B. Tasoglu S. (2021). Plant-based scaffolds in tissue engineering. ACS Biomater. Sci. Eng. 7 (3), 926–938. 10.1021/acsbiomaterials.0c01527 33591719

[B5] Böttcher-Haberzeth S. Biedermann T. (2019). Skin tissue engineering: Methods and protocols. New York, NYNew York: Springer.

[B6] Brandl F. P. Seitz A. K. Teßmar J. K. V. Blunk T. Göpferich A. M. (2010). Enzymatically degradable poly(ethylene glycol) based hydrogels for adipose tissue engineering. Biomaterials 31 (14), 3957–3966. 10.1016/j.biomaterials.2010.01.128 20170951

[B7] Bühler N. E. M. Schulze-Osthoff K. Königsrainer A. Schenk M. (2015). Controlled processing of a full-sized porcine liver to a decellularized matrix in 24 h. J. Biosci. Bioeng. 119 (5), 609–613. 10.1016/j.jbiosc.2014.10.019 25468420

[B8] Campiglio C. E. Contessi Negrini N. Farè S. Draghi L. (2019). Cross-linking strategies for electrospun gelatin scaffolds. Materials 12 (15), 2476. 10.3390/ma12152476 PMC6695673 31382665

[B9] Chan J. M. Zervantonakis I. K. Rimchala T. Polacheck W. J. Whisler J. Kamm R. D. (2012). Engineering of *in vitro* 3d capillary beds by self-directed angiogenic sprouting. PLoS ONE 7 (12), e50582. 10.1371/journal.pone.0050582 23226527 PMC3514279

[B10] Cheung H. K. Han T. T. Y. Marecak D. M. Watkins J. F. Amsden B. G. Flynn L. E. (2014). Composite hydrogel scaffolds incorporating decellularized adipose tissue for soft tissue engineering with adipose-derived stem cells. Biomaterials 35 (6), 1914–1923. 10.1016/j.biomaterials.2013.11.067 24331712

[B11] Colle J. Blondeel P. De Bruyne A. Bochar S. Tytgat L. Vercruysse C. (2020). Bioprinting predifferentiated adipose-derived mesenchymal stem cell spheroids with methacrylated gelatin ink for adipose tissue engineering. J. Mat. Sci. Mat. Med. 31 (4), 36. 10.1007/s10856-020-06374-w 32206922

[B12] Contessi Negrini N. Bonnetier M. Giatsidis G. Orgill D. P. Farè S. Marelli B. (2019). Tissue-mimicking gelatin scaffolds by alginate sacrificial templates for adipose tissue engineering. Acta Biomater. 87, 61–75. 10.1016/j.actbio.2019.01.018 30654214

[B13] Contessi Negrini N. Celikkin N. Tarsini P. Farè S. Święszkowski W. (2020). Three-dimensional printing of chemically crosslinked gelatin hydrogels for adipose tissue engineering. Biofabrication 12 (2), 025001. 10.1088/1758-5090/ab56f9 31715587

[B14] Contessi Negrini N. Toffoletto N. Farè S. Altomare L. (2020). Plant Tissues as 3D natural scaffolds for adipose, bone and tendon tissue regeneration. Front. Bioeng. Biotechnol. 8, 723. 10.3389/fbioe.2020.00723 32714912 PMC7344190

[B15] Czaja W. Krystynowicz A. Bielecki S. Brownjr R. (2006). Microbial cellulose—The natural power to heal wounds. Biomaterials 27 (2), 145–151. 10.1016/j.biomaterials.2005.07.035 16099034

[B16] Datta P. Ayan B. Ozbolat I. T. (2017). Bioprinting for vascular and vascularized tissue biofabrication. Acta Biomater. 51, 1–20. 10.1016/j.actbio.2017.01.035 28087487

[B17] Dikici S. Claeyssens F. MacNeil S. (2019). Decellularised baby spinach leaves and their potential use in tissue engineering applications: Studying and promoting neovascularisation. J. Biomater. Appl. 34 (4), 546–559. 10.1177/0885328219863115 31311391

[B18] Fitzgerald S. J. Cobb J. S. Janorkar A. V. (2020). Comparison of the formation, adipogenic maturation, and retention of human adipose‐derived stem cell spheroids in scaffold‐free culture techniques. J. Biomed. Mat. Res. 108 (7), 3022–3032. 10.1002/jbm.b.34631 PMC8506838 32396702

[B19] Fontana G. Gershlak J. Adamski M. Lee J. Matsumoto S. Le H. D. (2017). Biofunctionalized plants as diverse biomaterials for human cell culture. Adv. Healthc. Mat. 6 (8), 1601225. 10.1002/adhm.201601225 PMC5490445 28319334

[B20] Fung Y. C. Zweifach B. W. (1971). Microcirculation: Mechanics of blood flow in capillaries. Annu. Rev. Fluid Mech. 3 (1), 189–210. 10.1146/annurev.fl.03.010171.001201

[B21] Gershlak J. R. Hernandez S. Fontana G. Perreault L. R. Hansen K. J. Larson S. A. (2017). Crossing kingdoms: Using decellularized plants as perfusable tissue engineering scaffolds. Biomaterials 125, 13–22. 10.1016/j.biomaterials.2017.02.011 28222326 PMC5388455

[B22] Gibler P. Gimble J. Hamel K. Rogers E. Henderson M. Wu X. (2021). Human adipose-derived stromal/stem cell culture and analysis methods for adipose tissue modeling *in vitro*: A systematic review. Cells 10 (6), 1378. 10.3390/cells10061378 34204869 PMC8227575

[B23] Gilbert T. Sellaro T. Badylak S. (2006). Decellularization of tissues and organs. Biomaterials 27 (19), 3675. 10.1016/j.biomaterials.2006.02.014 16519932

[B24] Grolman J. M. Zhang D. Smith A. M. Moore J. S. Kilian K. A. (2015). Rapid 3D extrusion of synthetic tumor microenvironments. Adv. Mat. 27 (37), 5512–5517. 10.1002/adma.201501729 PMC4745120 26283579

[B25] Harris A. F. Lacombe J. Zenhausern F. (2021). The emerging role of decellularized plant-based scaffolds as a new biomaterial. Int. J. Mol. Sci. 22 (22), 12347. 10.3390/ijms222212347 34830229 PMC8625747

[B26] Hickey R. J. Modulevsky D. J. Cuerrier C. M. Pelling A. E. (2018). Customizing the shape and microenvironment biochemistry of biocompatible macroscopic plant-derived cellulose scaffolds. ACS Biomater. Sci. Eng. 4 (11), 3726–3736. 10.1021/acsbiomaterials.8b00178 33429594

[B27] Holland I. Logan J. Shi J. McCormick C. Liu D. Shu W. (2018). 3D biofabrication for tubular tissue engineering. Biodes. Manuf. 1 (2), 89–100. 10.1007/s42242-018-0013-2 30546921 PMC6267270

[B28] Huber B. Borchers K. Tovar G. E. Kluger P. J. (2016). Methacrylated gelatin and mature adipocytes are promising components for adipose tissue engineering. J. Biomater. Appl. 30 (6), 699–710. 10.1177/0885328215587450 26017717

[B29] Jansen K. Evangelopoulou M. Pou Casellas C. Abrishamcar S. Jansen J. Vermonden T. (2021). Spinach and chive for kidney tubule engineering: The limitations of decellularized plant scaffolds and vasculature. AAPS J. 23 (1), 11. 10.1208/s12248-020-00550-0 PMC7769781 33369701

[B30] Kessler L. Gehrke S. Winnefeld M. Huber B. Hoch E. Walter T. (2017). Methacrylated gelatin/hyaluronan-based hydrogels for soft tissue engineering. J. Tissue Eng. 8, 204173141774415. 10.1177/2041731417744157 PMC5753891 29318000

[B31] Kim J. B. Stein R. O’Hare M. J. (2004). Three-dimensional *in vitro* tissue culture models of breast cancer — A review. Breast Cancer Res. Treat. 85 (3), 281–291. 10.1023/b:brea.0000025418.88785.2b 15111767

[B32] Kolesky D. B. Homan K. A. Skylar-Scott M. A. Lewis J. A. (2016). Three-dimensional bioprinting of thick vascularized tissues. Proc. Natl. Acad. Sci. U. S. A. 113 (12), 3179–3184. 10.1073/pnas.1521342113 26951646 PMC4812707

[B33] Laschke M. W. Menger M. D. (2016). Prevascularization in tissue engineering: Current concepts and future directions. Biotechnol. Adv. 34 (2), 112–121. 10.1016/j.biotechadv.2015.12.004 26674312

[B34] Lim K. S. Schon B. S. Mekhileri N. V. Brown G. C. J. Chia C. M. Prabakar S. (2016). New visible-light photoinitiating system for improved print fidelity in gelatin-based bioinks. ACS Biomater. Sci. Eng. 2 (10), 1752–1762. 10.1021/acsbiomaterials.6b00149 33440473

[B35] Lopes S. V. Collins M. N. Reis R. L. Oliveira J. M. Silva-Correia J. (2021). Vascularization approaches in tissue engineering: Recent developments on evaluation tests and modulation. ACS Appl. Bio Mat. 4 (4), 2941–2956. 10.1021/acsabm.1c00051 35014385

[B36] Lovett M. Lee K. Edwards A. Kaplan D. L. (2009). Vascularization strategies for tissue engineering. Tissue Eng. Part B Rev. 15 (3), 353–370. 10.1089/ten.teb.2009.0085 19496677 PMC2817665

[B37] Lugert S. Unterweger H. Mühlberger M. Janko C. Draack S. Ludwig F. (2018). Cellular effects of paclitaxel-loaded iron oxide nanoparticles on breast cancer using different 2D and 3D cell culture models. Int. J. Nanomedicine 14, 161–180. 10.2147/ijn.s187886 30613144 PMC6306067

[B38] Mahmoudzadeh A. Mohammadpour H. (2016). Tumor cell culture on collagen–chitosan scaffolds as three-dimensional tumor model: A suitable model for tumor studies. J. Food Drug Anal. 24 (3), 620–626. 10.1016/j.jfda.2016.02.008 28911569 PMC9336670

[B39] Mandrycky C. Wang Z. Kim K. Kim D. H. (2016). 3D bioprinting for engineering complex tissues. Biotechnol. Adv. 34 (4), 422–434. 10.1016/j.biotechadv.2015.12.011 26724184 PMC4879088

[B40] McCarthy M. Brown T. Alarcon A. Williams C. Wu X. Abbott R. D. (2020). Fat-on-a-chip models for research and discovery in obesity and its metabolic comorbidities. Tissue Eng. Part B Rev. 26 (6), 586–595. 10.1089/ten.teb.2019.0261 32216545 PMC8196547

[B41] Miller J. S. Stevens K. R. Yang M. T. Baker B. M. Nguyen D. H. T. Cohen D. M. (2012). Rapid casting of patterned vascular networks for perfusable engineered three-dimensional tissues. Nat. Mat. 11 (9), 768–774. 10.1038/nmat3357 PMC3586565 22751181

[B42] Modulevsky D. J. Cuerrier C. M. Pelling A. E. (2016). Biocompatibility of subcutaneously implanted plant-derived cellulose biomaterials. PLoS One 11 (6), e0157894. 10.1371/journal.pone.0157894 27328066 PMC4915699

[B43] Mohiuddin O. A. O’Donnell B. T. Poche J. N. Iftikhar R. Wise R. M. Motherwell J. M. (2019). Human adipose-derived hydrogel characterization based on *in vitro* asc biocompatibility and differentiation. Stem Cells Int. 2019, 1–13. 10.1155/2019/9276398 PMC7012213 32082388

[B44] Müller F. A. Müller L. Hofmann I. Greil P. Wenzel M. M. Staudenmaier R. (2006). Cellulose-based scaffold materials for cartilage tissue engineering. Biomaterials 27 (21), 3955–3963. 10.1016/j.biomaterials.2006.02.031 16530823

[B45] Nichol J. W. Koshy S. T. Bae H. Hwang C. M. Yamanlar S. Khademhosseini A. (2010). Cell-laden microengineered gelatin methacrylate hydrogels. Biomaterials 31 (21), 5536–5544. 10.1016/j.biomaterials.2010.03.064 20417964 PMC2878615

[B46] Ogushi Y. Sakai S. Kawakami K. (2013). Adipose tissue engineering using adipose-derived stem cells enclosed within an injectable carboxymethylcellulose-based hydrogel: Adipose tissue engineering using injectable hydrogel. J. Tissue Eng. Regen. Med. 7 (11), 884–892. 10.1002/term.1480 22489051

[B47] Ott H. C. Matthiesen T. S. Goh S. K. Black L. D. Kren S. M. Netoff T. I. (2008). Perfusion-decellularized matrix: Using nature’s platform to engineer a bioartificial heart. Nat. Med. 14 (2), 213–221. 10.1038/nm1684 18193059

[B48] Ovsianikov A. Deiwick A. Van Vlierberghe S. Pflaum M. Wilhelmi M. Dubruel P. (2011). Laser fabrication of 3D gelatin scaffolds for the generation of bioartificial tissues. Materials 4 (1), 288–299. 10.3390/ma4010288 28879989 PMC5448471

[B49] Paek J. Park S. E. Lu Q. Park K. T. Cho M. Oh J. M. (2019). Microphysiological engineering of self-assembled and perfusable microvascular beds for the production of vascularized three-dimensional human microtissues. ACS Nano 13 (7), 7627–7643. 10.1021/acsnano.9b00686 31194909

[B50] Rijal G. Li W. (2016). 3D scaffolds in breast cancer research. Biomaterials 81, 135–156. 10.1016/j.biomaterials.2015.12.016 26731577

[B51] Ryu N. E. Lee S. H. Park H. (2019). Spheroid culture system methods and applications for mesenchymal stem cells. Cells 8 (12), 1620. 10.3390/cells8121620 PMC6953111 31842346

[B52] Saberianpour S. Heidarzadeh M. Geranmayeh M. H. Hosseinkhani H. Rahbarghazi R. Nouri M. (2018). Tissue engineering strategies for the induction of angiogenesis using biomaterials. J. Biol. Eng. 12 (1), 36. 10.1186/s13036-018-0133-4 30603044 PMC6307144

[B53] Sims-Mourtada J. Niamat R. Samuel S. Eskridge C. Kmiec E. (2014). Enrichment of breast cancer stem-like cells by growth on electrospun polycaprolactone-chitosan nanofiber scaffolds. Int. J. Nanomedicine 995, 995–1003. 10.2147/IJN.S55720 PMC3933718 24570583

[B54] Song H. H. G. Park K. M. Gerecht S. (2014). Hydrogels to model 3D *in vitro* microenvironment of tumor vascularization. Adv. Drug Deliv. Rev. 79–80, 19–29. 10.1016/j.addr.2014.06.002 PMC4258430 24969477

[B55] Sullivan K. E. Quinn K. P. Tang K. M. Georgakoudi I. Black L. D. (2014). Extracellular matrix remodeling following myocardial infarction influences the therapeutic potential of mesenchymal stem cells. Stem Cell Res. Ther. 5 (1), 14. 10.1186/scrt403 24460869 PMC4055039

[B56] Thippan M. Dhoolappa M. Lakshmishree K. Sheela P. Prasad R. (2019). Morphology of medicinal plant leaves for their functional vascularity: A novel approach for tissue engineering applications. Int. J. Chem. Stud. 7 (3), 55–58.

[B57] Toker M. Rostami S. Kesici M. Gul O. Kocaturk O. Odabas S. (2020). Decellularization and characterization of leek: A potential cellulose-based biomaterial. Cellulose 27 (13), 7331–7348. 10.1007/s10570-020-03278-4

[B58] Tomasina C. Bodet T. Mota C. Moroni L. Camarero-Espinosa S. (2019). Bioprinting vasculature: Materials, cells and emergent techniques. Materials 12 (17), 2701. 10.3390/ma12172701 PMC6747573 31450791

[B59] Tsao C. T. Kievit F. M. Wang K. Erickson A. E. Ellenbogen R. G. Zhang M. (2014). Chitosan-based thermoreversible hydrogel as an *in vitro* tumor microenvironment for testing breast cancer therapies. Mol. Pharm. 11 (7), 2134–2142. 10.1021/mp5002119 24779767 PMC4096230

[B60] Varhama K. Oda H. Shima A. Takeuchi S. (2019). “Decellularized plant leaves for 3d cell culturing,” in IEEE 32nd International Conference on Micro Electro Mechanical Systems (MEMS), Seoul, Korea (South), 27-31 January 2019 (IEEE). 10.1109/MEMSYS.2019.887062 226–8

[B61] Vinson B. T. Phamduy T. B. Shipman J. Riggs B. Strong A. L. Sklare S. C. (2017). Laser direct-write based fabrication of a spatially-defined, biomimetic construct as a potential model for breast cancer cell invasion into adipose tissue. Biofabrication 9 (2), 025013. 10.1088/1758-5090/aa6bad 28382922

[B62] Wang Y. Dominko T. Weathers P. J. (2020). Using decellularized grafted leaves as tissue engineering scaffolds for mammalian cells. Vitro Cell. Dev. Biol. -Plant. 56 (6), 765–774. 10.1007/s11627-020-10077-w

[B63] Wang Z. Abdulla R. Parker B. Samanipour R. Ghosh S. Kim K. (2015). A simple and high-resolution stereolithography-based 3D bioprinting system using visible light crosslinkable bioinks. Biofabrication 7 (4), 045009. 10.1088/1758-5090/7/4/045009 26696527

[B64] Yang F. Carmona A. Stojkova K. Garcia Huitron E. I. Goddi A. Bhushan A. (2021). A 3D human adipose tissue model within a microfluidic device. Lab. Chip 21 (2), 435–446. 10.1039/d0lc00981d 33351023 PMC7876365

[B65] Zaborowska M. Bodin A. Bäckdahl H. Popp J. Goldstein A. Gatenholm P. (2010). Microporous bacterial cellulose as a potential scaffold for bone regeneration. Acta Biomater. 6 (7), 2540–2547. 10.1016/j.actbio.2010.01.004 20060935

[B66] Zhang S. Liu P. Chen L. Wang Y. Wang Z. Zhang B. (2015). The effects of spheroid formation of adipose-derived stem cells in a microgravity bioreactor on stemness properties and therapeutic potential. Biomaterials 41, 15–25. 10.1016/j.biomaterials.2014.11.019 25522961

[B67] Zhang Y. S. Duchamp M. Oklu R. Ellisen L. W. Langer R. Khademhosseini A. (2016). Bioprinting the cancer microenvironment. ACS Biomater. Sci. Eng. 2 (10), 1710–1721. 10.1021/acsbiomaterials.6b00246 28251176 PMC5328669

