# Supplementary material for: Decellularized fennel and dill leaves as possible 3D channel network in GelMA for the development of an in vitro adipose tissue model
Source: Front Bioeng Biotechnol. 2022 Oct 31;10:984805. doi: 10.3389/fbioe.2022.984805 (PMC9659726; doi:10.3389/fbioe.2022.984805)

Decellularized fennel and dill leaves as possible 3D channel network in GelMA for the development of an in vitro adipose tissue model

Supplementary Material

Supplementary Figure S1 – Channel size distribution for the decellularized leaves: in the upper row, the detail of the area where the cross-section was observed is reported; in the middle row, a SEM image of the cross-section of the D-leaf; in the third row the distribution of the diameter of the channels obtained by ImageJ is reported for each D-leaf.

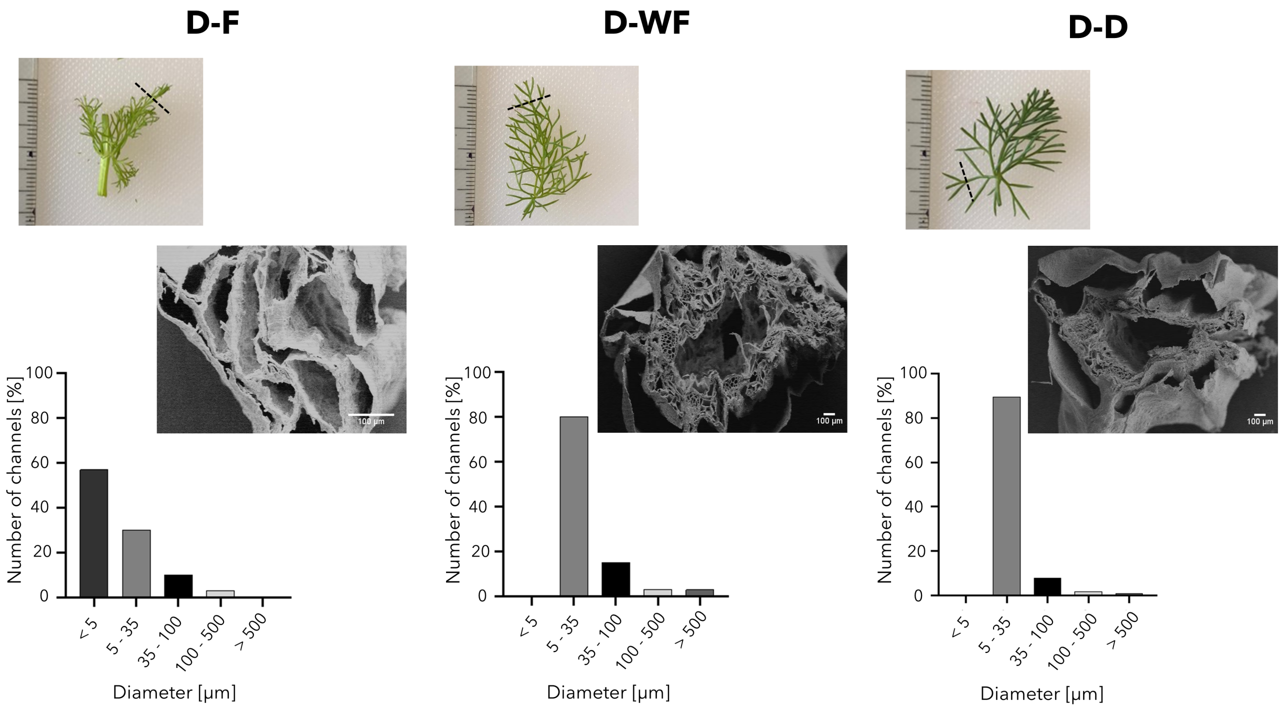

Supplementary Material 2

Gel fraction

Method

Gel fraction, representative of the percentage of the solid fraction of the hydrogel at the considered time point, was calculated. Crosslinked samples (n = 3) of GelMA/D-F and GelMA were analyzed. Samples were dehydrated by immersion, immediately after crosslinking, in ethanol for 24 h, and weighed (w_a). Then, samples were immersed in an aqueous solution with 0.02% w/v NaN_3 at 37 °C, then at 24 and 72 h of incubation, they were frozen at -80 °C, freeze-dried and weighed (w_g). The gel fraction percentage value (*GF %*) was calculated using Equation (S1):

$$GF\ [\%] = \frac{w_g}{w_a} \times 100 \quad \text{(S1)}$$

Results

The percentage of the solid gel fraction in the GelMA/D-F structure, representative of the three GelMA/D-leaf structures, was evaluated in order to verify the efficiency of the crosslinking by Irgacure 2959 and exposure to UV rays, in presence of 3D decellularized plant embedded in the hydrogel. The gel fraction of pristine crosslinked GelMA was considered for comparison. The analysis was carried out at two different time points (i.e., 24 and 72 h), to verify the trend of the possible material loss over the time. The timepoints were selected considering the results of the stability tests: at 24 h all the samples reached the minimum weight ($p > 0.05$), and at 72 h before reaching the swelling plateau. In Figure S1 gel fraction values for the two considered structures are reported. No statistically significant differences were detected between the two formulations for both the timepoints. This result confirms the weight variation trends, showing no influence of the embedded decellularized leaf in the GelMA hydrogel during the photo-crosslinking process. After 24 h of incubation, upon reaching their minimum weight, the fraction gel value for the GelMA/D-F formulation is about 75%, while for GelMA it turns out about 80%. After 72 h of swelling, upon reaching the absorption plateau, the value of gel fraction for the formulation of GelMA/D-F is about 75% and for the GelMA it is about 80%, remaining unchanged from the previous time point. These results demonstrated that the photo-crosslinking process was equally efficient for the considered formulations. After an initial material loss, caused by the leakage of non-crosslinked gelatin chains, GelMA did not show any further weight loss, demonstrating no significant degradation, and no influence of the 3D decellularized structure on the crosslinking procedure.

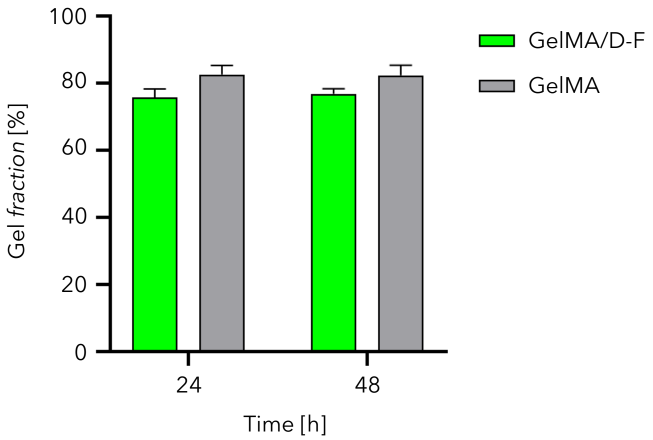

Supplementary Figure S2 - Gel fraction [%] after 24 and 72 h of incubation for crosslinked pristine GelMA and GelMA/D-F.

Supplementary Table S1 - Dimensions of the microchannels detected in the decellularized leaf structures, measured by ImageJ, starting from images obtained at SEM at 1000X magnification. The obtained data are compared to the average size of human microcirculation.

**Channels**	**Diameter [μm]**
Human arterioles [SM1]	30 μm
Human capillaries [SM1]	5 - 10 μm
D-F	3 - 20 μm
D-WF	5 - 35 μm
D-D	5 - 35 μm

SM1 YC Fung YC, BW Zweifach. Microcirculation: Mechanics of Blood Flow in Capillaries. Annual Review of Fluid Mechanics. 1971;3:189-210.

Supplementary Figure S3 – Mechanical parameters obtained by compression test on crosslinked GelMA and GelMA/D-leaf: A) elastic modulus [kPa], B) stiffness [kPa], C) maximum stress [kPa], D) residual strain [%], E) hysteresis area [J/dm^3]. *: $p < 0.05$, **: $p < 0.01$, *** $p < 0.001$.

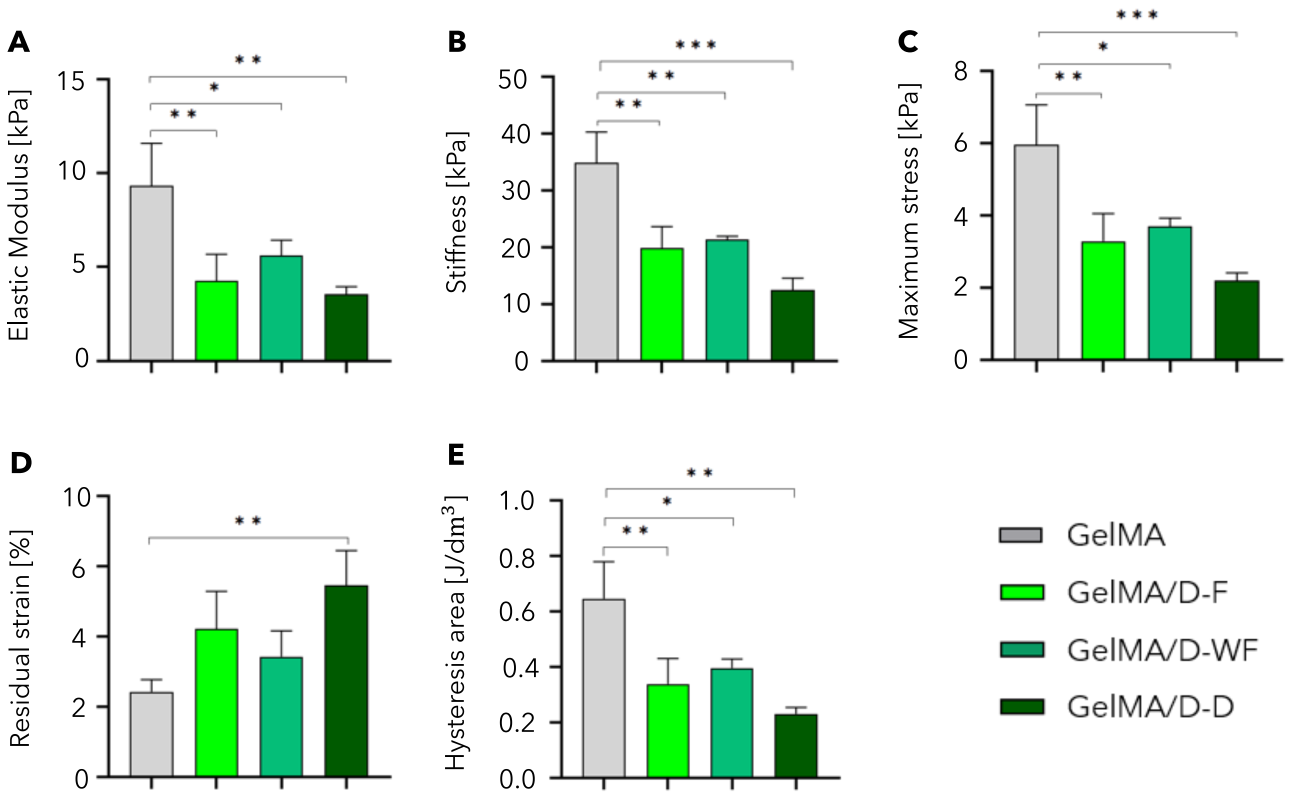

Supplementary Figure S4 – Live/Dead staining on GelMA/D-leaf structures after 1, 3, and 7 days of culture. For each considered culture timepoint, two representative images are reported: on the left a zone of the sample close to the D-leaf, and on the right a zone far from the D-leaf. The dotted line highlights the D-leaf (in yellow) or the perimeter (in blue) of the sample. Scale bar: 250 µm.

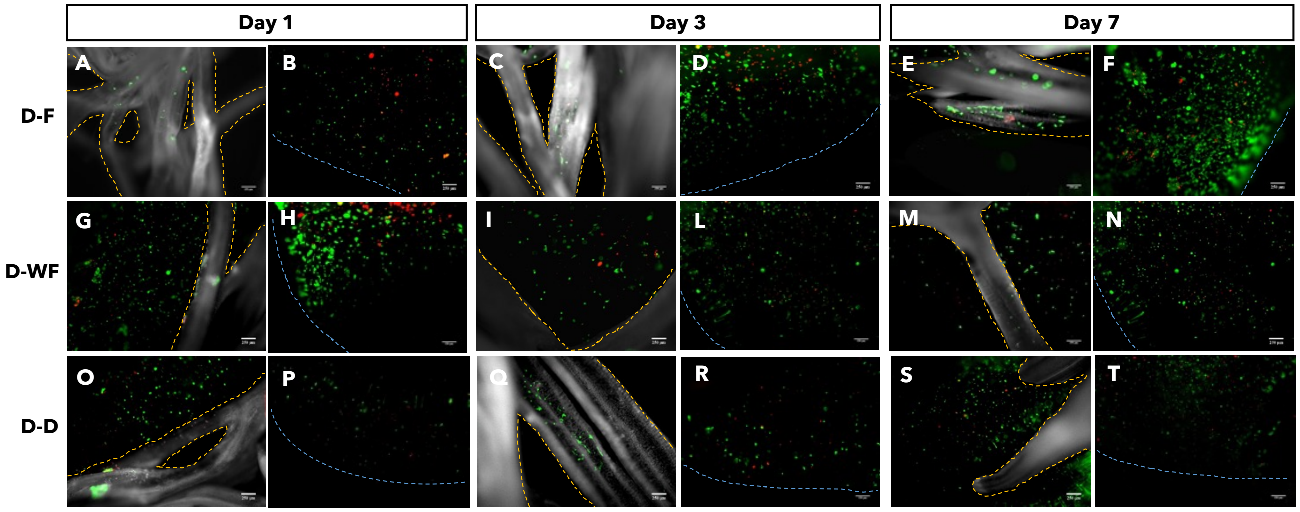

Supplementary Video S1 – Perfusion test on GelMA/D-F structures by injection of red food colouring.

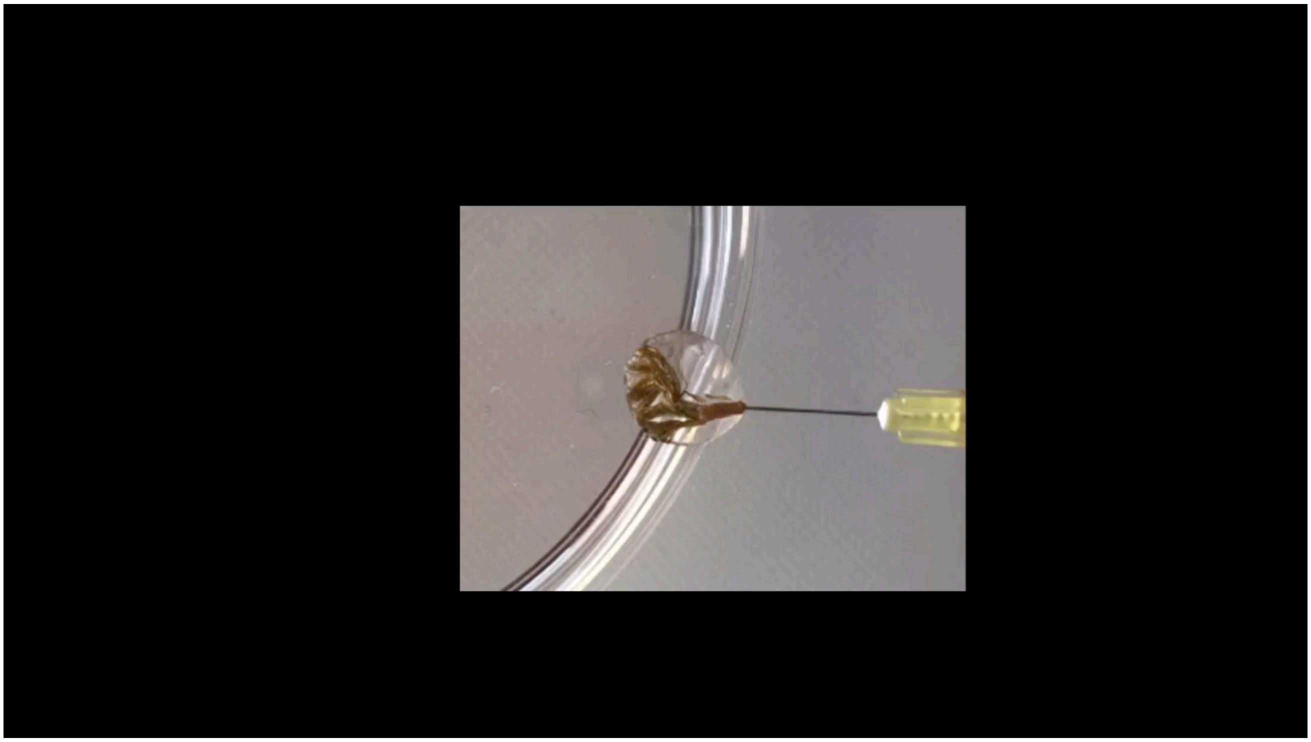

Supplement: Supplementary file 1 [file Presentation1.pdf]
